# A systematic review and meta-analysis of 24-h urinary output of children and adolescents: impact on the assessment of iodine status using urinary biomarkers

**DOI:** 10.1007/s00394-019-02151-w

**Published:** 2019-11-29

**Authors:** Kelsey Beckford, Carley A. Grimes, Claire Margerison, Lynn J. Riddell, Sheila A. Skeaff, Madeline L. West, Caryl A. Nowson

**Affiliations:** 1 Institute for Physical Activity and Nutrition, Deakin University, Locked Bag 20000, Waurn Ponds, Geelong, VIC 3220 Australia; 2 Department of Human Nutrition, University of Otago, 362 Leith St, North Dunedin, Dunedin, 9016 New Zealand

**Keywords:** Iodine, Urine, Urine volume, Children, Adolescents, Nutrition, Assessment

## Abstract

**Purpose:**

Urinary iodine concentration (UIC (μg/ml) from spot urine samples collected from school-aged children is used to determine the iodine status of populations. Some studies further extrapolate UIC to represent daily iodine intake, based on the assumption that children pass approximately 1 L urine over 24-h, but this has never been assessed in population studies. Therefore, the present review aimed to collate and produce an estimate of the average 24-h urine volume of children and adolescents (> 1 year and < 19 years) from published studies.

**Methods:**

EBSCOHOST and EMBASE databases were searched to identify studies which reported the mean 24-h urinary volume of healthy children (> 1 year and < 19 years). The overall mean (95% CI) estimate of 24-h urine volume was determined using a random effects model, broken down by age group.

**Results:**

Of the 44 studies identified, a meta-analysis of 27 studies, with at least one criterion for assessing the completeness of urine collections, indicated that the mean urine volume of 2–19 year olds was 773 (654, 893) (95% CI) mL/24-h. When broken down by age group, mean (95% CI) 24-h urine volume was 531 mL/day (454, 607) for 2–5 year olds, 771 mL/day (734, 808) for 6–12 year olds, and 1067 mL/day (855, 1279) for 13–19 year olds.

**Conclusions:**

These results demonstrate that the average urine volume of children aged 2–12 years is less than 1 L, therefore, misclassification of iodine intakes may occur when urine volumes fall below or above 1 L. Future studies utilizing spot urine samples to assess iodine status should consider this when extrapolating UIC to represent iodine intakes of a population.

**Electronic supplementary material:**

The online version of this article (10.1007/s00394-019-02151-w) contains supplementary material, which is available to authorized users.

## Introduction

Monitoring the iodine nutrition of populations and individuals is important to identify those at risk of deficiency, as deficiencies during childhood have been linked with impaired cognitive and motor functions in schoolchildren [1, 2]. Current recommendations for assessing the iodine nutrition of populations using spot urine samples from school-aged children were developed by the World Health Organization (WHO) over a number of years (1986–2007) [3–7]. Whilst the original version of these recommendations was based on the association between a daily estimated iodine intake, extrapolated from creatinine excretion, equivalent to 100 µg of iodine and reduced goiter prevalence among children and adults [8], later iterations extrapolated this value to represent a concentration, urinary iodine concentration (UIC), expressed as µg/L [6]. Whilst these recommendations were originally developed as a marker of iodine status within populations, some studies have utilized UIC from spot urine samples as an indicator of iodine intake [9–14]. In such instances, issues may arise as UIC would only be reflective of daily intake if urine volume was equivalent to 1 L. For example, in a population with a daily urinary volume of 1 L, a UIC of 100 µg/L could be extrapolated to be indicative of a daily iodine intake of 100 µg/24-h. As this value is equal to the daily intake originally associated with reduced goiter prevalence [8], this population can be classified as having a sufficient iodine intake. However, in a population of children with a daily urine volume closer to 0.5 L, a UIC of 100 µg/L may be indicative of a daily iodine intake closer to 50 µg/24-h. This could result in the misclassification of this population as iodine sufficient when their daily iodine intake may, in fact, place them at risk of developing iodine deficiency disorders.

Therefore, iodine monitoring programs which have extrapolated daily iodine intake from UIC determined from spot urine samples in populations of children and adolescents [9–14] and have not taken the lower daily urinary output into account, may be inaccurately estimating iodine intakes. As such, this may have resulted in the misclassification of populations as having sufficient iodine intakes, when their true intakes may be lower than the 100 µg/day originally associated with reduced goiter prevalence. Such misclassifications may have prevented the implementation of necessary iodine fortification programs. To date, there has been no global systematic collation of the average 24-h urine volume of children and adolescents. This information could help researchers estimate population dietary iodine intake of children and adolescents from spot urine samples. Therefore, the aim of the current study was to estimate the average 24-h urine volume measured in healthy children and adolescents, by conducting a systematic review and meta-analysis of studies which have reported the 24-h urine volume of children and adolescents aged 2–19 years.

## Methods

This protocol adheres to the Preferred Reporting Items for Systematic Review and Meta-Analysis Protocols (PRISMA-P) 2015 statement [15] and was registered with the International Prospective Register of Systematic Reviews (PROSPERO) (registration number CRD42016033682).

### Information sources and search

A search strategy was developed to identify papers published up to October 2018, which have reported the 24-h urine volume of children and adolescents (> 1 year and ≤ 19 years). An electronic literature search of EBSCOHOST (MEDLINE complete, CINAHL, Academic Search Complete and Global Health) and EMBASE databases was conducted. The search strategy was developed in consultation with a research librarian. Free text keywords were used to conduct the search. Search criteria specific to each database are outlined in Table 1. The search strategy was piloted across each database to improve the effectiveness of the final search. Only peer-reviewed original research articles published in English and conducted in humans were included. It was beyond the scope of this review to include and examine sources from ‘grey’ literature. The reference lists of included studies identified through the search were also reviewed.

**Table 1:** Search criteria specifications for each database

Database	Search options	Search terms
EBSCOHOST		
Academic search complete	Limiters—full text; scholarly (peer reviewed) journals; language: English Search modes—boolean/phrase	(“24 h urin*” OR “twenty four hour urin*” OR “24 h urin*”) AND (sampl* OR collection* OR volume* OR excretion* OR output*) AND (schoolchild* OR child* OR adolescen* OR teen*)
CINAHL complete	Limiters—English language; peer reviewed Search modes—boolean/phrase	
Global health	Limiters—language: English Search modes—boolean/phrase	
MEDLINE complete	Limiters—English language; human Search modes—boolean/phrase	
EMBASE	Advanced search No mapping options used No date limits specified Sources: Embase only (Medline not selected as separate search) Field labels: abstract, article title, index term and subheading Quick limits: human, only in English Publication types: article, article in press EBM, gender, age and animal advanced options left blank	#1: ‘24 h urin*’ OR ‘twenty four hour urin*’ OR ‘24 h urin*’ OR ‘twenty four h urin*’ #2: sampl* OR collection* OR volume* OR excretion*or AND output* #3: child* OR adolescen* OR teen* #4: #1 AND #2 AND #3

### Inclusion/exclusion criteria

Only peer-reviewed original research studies were included and any reviews, meta-analyses, editorials, case reports, conference proceedings or other grey literature identified through the search were excluded at the screening stage. As the primary focus of this review was to determine the average daily urinary output of children and adolescents, only studies which reported the 24-h urinary output of healthy children and adolescents > 1 and ≤ 19 years of age were included. Where multiple published reports were available from the same study, the most recently published and/or the study with the largest sample size was included.

**Fig. 1:**
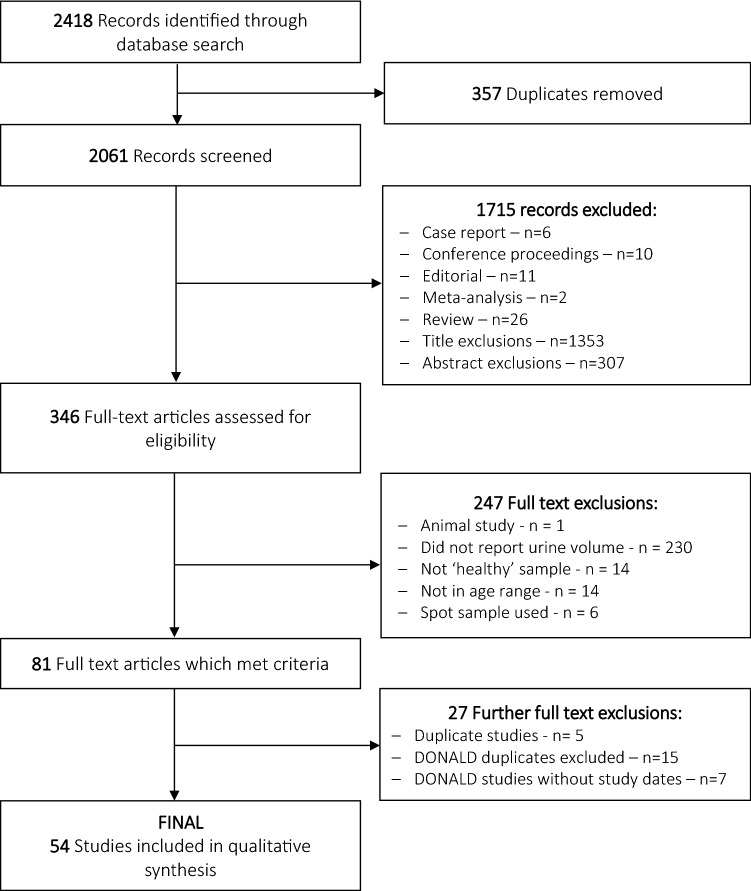
PRISMA flowchart

All papers identified from the initial electronic search process were imported into an Endnote library, and duplicates removed. Titles and abstracts were screened and studies included based on the eligibility criteria as outlined above. Two investigators (KB and MW) independently screened the titles and abstracts of the articles independently to assess eligibility for inclusion. If agreement was reached, articles were either excluded or moved to the next stage (full-text). If agreement was not reached, the article was moved to the full-text stage. Following this screening process, the full text of eligible studies was retrieved and studies which collected 24-h urine samples but did not report the final 24-h urine volume were excluded. At this stage, the reference lists of included studies were scanned, and the full text of any relevant studies retrieved and reviewed for inclusion. The PRISMA flow chart [16] was used to document the number of studies identified during the search process and those excluded and included according to the outlined eligibility criteria (Fig. 1).

As multiple studies had utilised data from the Dortmund Nutritional and Anthropometric Longitudinally Designed (DONALD) Study [17], cross-checking of study dates and participant characteristics was carried out to minimise participant overlap between the studies. Some studies (*n* = 7) [18–24] were excluded from the final analysis as they did not report which years of data collection had been analysed, therefore, possible participant overlap with other studies could not be determined. No additional information apart from that published was available from authors. Where participant overlap was possible, the study with the larger participant number was included in the final analysis. Of the 27 DONALD studies initially identified, six studies [25–30] were considered for the final analyses as they captured the full range of data collection years and included the largest number of participants whilst minimising possible participant overlap.

### Data extraction and synthesis

Data extraction was completed using a data extraction template (Table 2). The template was initially piloted on five eligible studies and modifications made where necessary. As 24-h urinary creatinine excretion either alone, in relation to expected creatinine based on sex and/or weight, is often used as a marker for complete urine collection under the assumption that urinary creatinine excretion, as an indicator of body mass is stable within individuals from day to day [31–33], data pertaining to 24-h excretion of creatinine was also extracted where reported.

**Table 2:** Data extracted from included studies

Study overview
Aim(s) Study design Study year(s) Description of country where the study was conducted Season of the year Setting (i.e., home/school)
Methods
Recruitment method Inclusion criteria (main study—not urine specific) Specific exclusion criteria (main study—not urine specific) Data collection overview
24-h urine collection protocol
24-h urine protocol description Criteria for completeness of urine samples - Time of collection (hours) - Volume of collection - Creatinine cut-off - Number of missed collections reported by child/parent Specific exclusion criteria for urine samples Adjustment for collection time/normalised? Other nutrients analysed
Participant characteristics
Total no. participating No. exclusions No. withdrawals Final *n* included in analyses % male participants Total no. urine samples Age, mean and range Weight category/BMI Race/ethnicity SES (include description of SES definition)
Results
Urine volume (L/24 h)—mean, SD/SEM, median, range Creatinine—mean, SD/SEM, median, range

### Quality assessment

The quality of the studies included in this review was assessed using a modified version of the Newcastle–Ottawa scale (NOS) for cohort studies [34], as all studies included in the final synthesis were of a cross-sectional study design. The NOS was modified to suit the context of the studies included in the review and particular consideration was made towards the 24-h urine collection methods used in each study. This scale assigns stars to indicate higher quality based on three broad criteria specific to the design of the study: (1) selection (representativeness of the study sample); (2) comparability of the findings (normalisation of the results to a 24-h period); and (3) assessment of outcome (quality of the reported 24-h urine collection methodology) (Online Supporting Material). Studies were categorised as ‘high’ ‘medium’ or ‘low’ quality according to the number of stars they received (out of a maximum of 10 stars: low: 0–3; medium: 4–7; high 8–10). As only three studies provided sufficient detail on their urine collection protocol to be classified as “high” quality [35–37], we included a second category of quality assessment, based on studies which had reported at least one criterion for the assessment of urine collection completeness.

### Statistical analysis

**Fig. 2:**
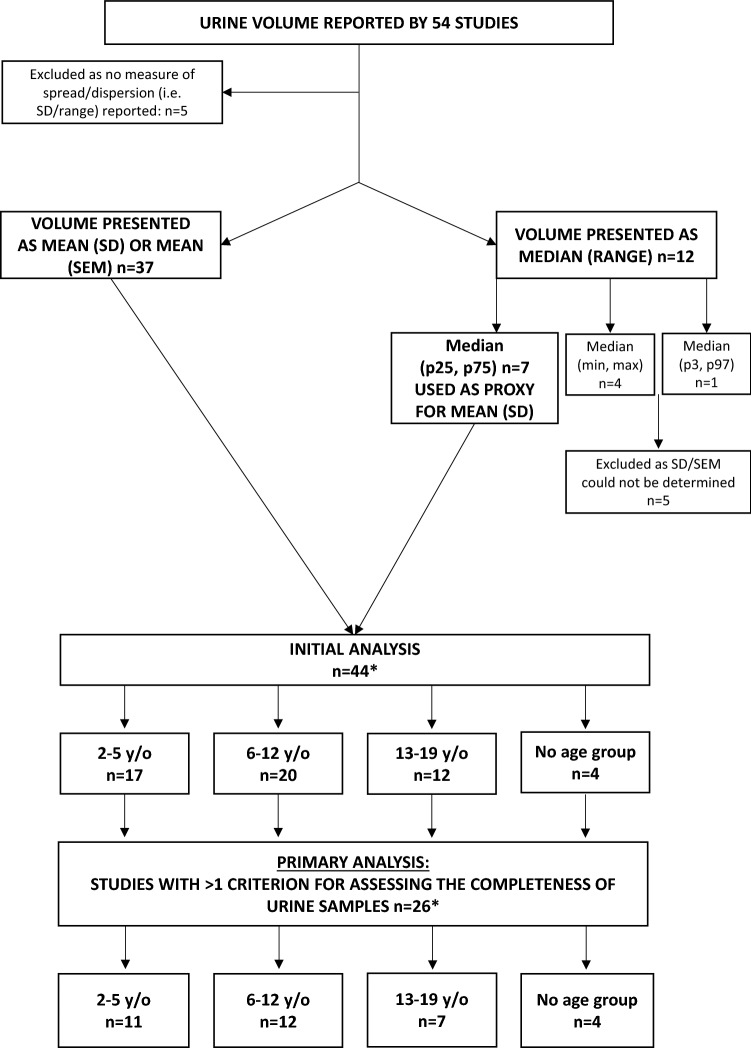
Flow chart of studies included in analyses

Following data extraction, data was collated and imported into STATA/SE 15.0 (StataCorp LP, College Station, TX, USA) for analysis. The main outcome variable was 24-h urine volume, presented in mL/24-h. Of the 54 studies originally included, five did not include a measure of spread/dispersion [38–42] and were subsequently excluded (Fig. 2). Most studies (*n* = 37) reported urine volume as mean (SD) or mean (SEM) [35–37, 43–76] (Table 3). Twelve studies reported urine volume as median, this included median (min, max) *n* = 4 [77–80], median (IQR) *n* = 7 [25, 26, 28–30, 81, 82], median (P3, P97) *n* = 1 [83]. For the seven studies which reported median (IQR) (Table 4), the mean (SD) was extrapolated from the median (IQR) using the median as a proxy for the mean and the IQR as a proxy for the SD (i.e., P75–P25 = SD) [84]. The calculated mean (SD) for these studies was then pooled with the results of those studies which reported urine volume as mean (SD). As such, a total of 44 studies were considered for the primary analysis.

**Table 3:** Characteristics of studies considered for primary analysis where 24-h urine volume was presented as mean (SD/SEM), *n* = 37 studies

Study	Study Year(s)	*n*	Male, *n* (%)	Valid urine samples	Age range (years)	Age, mean (SD) years	Country	Exclusion criteria for urine samples	NOS score	
									Score out of 10	Rating*
Abuhaloob [43]	Not described	216	104 (48%)	216	3–5	4.1 (0.3)	Gaza strip	(1) Urine flow rate < 140 ml in 24 h and (2) Urinary creatinine concentration < 0.1 or > 1.5 mg/ml	5	Medium
Acevedo [44]	Not described	32	Not described	32	3–5	4.1 (0.8)	Caracas, Venezuela	Not described	2	Low
		31		31	3–5	4.3 (1.3)	San Juan de los Morros, Venezuela			
Allevard-Burguburu [45]	Not described	7	Not described	7	2–2.9	Not described	Not described	Not described	0	Low
		13		13	3–4.9					
		16		16	5–6.9					
		16		16	7–8.9					
		10		10	9–11					
Aparicio [46]	2014	205	109 (53%)	205	7–11	8.8 (1.2)	Spain	Creatinine excretion < 0.1 mmol/kg/day	0	Low
Ballauff [47]	Not described	21	10 (48%)	21	6–11	8.8 (1.4)	Not described	Not described	4	Medium
Clark [48]	Not described	204	89 (47%)	190	9–10	9.6 (0.2)	Salisbury, United Kingdom	“Fourteen urine samples were excluded because of an incorrect collection period (*n* = 4), a urine volume almost 4 standard deviations below the geometric mean (*n* = 1), or bacterial or other contamination (*n* = 9)”	4	Medium
Fujishima [49]	1980	84	84 (100%)	76	15–18	Not described	Kanagawa, Japan	Not described	2	Low
Grimes [35]	2010 –2013	168	96 (57%)	168	4–7.9	6.9 (0.7)	Victoria, Australia	One or more of the following criteria: (1) Collection time < 20 or > 28 h (2) Total volume < 300 ml (3) Participant reported missing > 1 collection, or (4) Urinary creatinine excretion < 0.1 mmol/kg/day	10	High
		498	269 (54%)	498	8–12	10.1 (1.2)				
Haftenberger [50]	1998	11	6 (55%)	11	3–6	4.2 (1.3)	Dresden, Germany	Not described	0	Low
Haga [51]	2005	29	13 (45%)	29	3–4	Not described	Miyagi, Japan	Not described	2	Low
		33	18 (55%)	33	4.1–5					
		17	8 (47%)	17	5.1–6					
He [52]	2013	138	67 (49%)	138	9–11	10.2 (0.5)	Changzhi, China	(1) Urine volume < 300 ml/24-h or (2) Creatinine < 5th centile (< 2.5 mmol/24-h for girls and < 2.9 mmol/24-h for boys)	7	Medium
		141	67 (48%)	141	9–12	10.0 (0.5)				
Hesse [53]	Not described	25	25 (100%)	25	15–19	Not described	Not described	Not described	0	Low
		25	0 (0%)	25	15–19					
Höllriegl [54]	2008	9	Not described	8	3–5	Not described	Akure, Nigeria	Urinary creatinine values outside of the range of 0.2–3.0 g/L	3	Low
		28		24	6–10					
		12		5	11–15					
Juarez-Lopez [55]	Not described	20	30 (54%)	20	3–5	3.8 (0.9)	State of Mexico	Excretion rate below 9 μg F/h	3	Low
Ketley [57]	Not described	13	Not described	65	5–6	5.8 (0.5)	Merseyside, England	Urinary flow rate < 9 mL/h	3	Low
Ketley [56]	1994–1996	19	Not described	19	1.5–3.5	3.4 (0.2)	Cork, Ireland	Urinary fl̄ow rate of < 9 ml/h or > 420 ml/h	6	Medium
		18		18	1.5–3.5	3.1 (0.6)	Knowsley, England			
		18		18	1.5–3.5	3.1 (0.4)	Oulu, Finland			
		4		4	1.5–3.5	3.6 (0.3)	Reykjavik, Iceland			
		6		6	1.5–3.5	3.2 (0.5)	Haarlem, the Netherlands			
		21		21	1.5–3.5	3.1 (0.4)	Almada/Setubal, Portugal			
Khandare [58]	Not described	18	18 (100%)	18	9–11	10.7 (1.4)	Andhra Pradesh, India	Not described	0	Low
Kirdpon [59]	Not described	13	13 (100%)	13	9–13	12.4 (0.3)	Not described	Not described	0	Low
Kristbjornsdottir [60]	Not described	76	30 (52%)	58	5–6	6 (0.1)	Reykjavik, Iceland	PABA recovery < 85%	5	Medium
Maguire [61]	Not described	12	Not described	12	6–7	6.6 (0.3)	Northern England	(1) Urinary flow < 5 ml/h for < 6 year olds and < 9 mL/h for children ≥ 6 years (2) Creatinine excretion < 11.3 mg/kg/day or > 21.1 mg/kg/day	7	Medium
		11		11	6–7	6.6 (0.5)				
		13		13	6–7	7.0 (0.7)				
Marrero [36]	2007–2010	150	61 (52%)	126	5–6	5.5 (0.6)	London, England	(1) The participant admitted to have missed at least one urine collection (2) 24-h urinary creatinine of < 0.1 mmol/kg/d (3) urine output of < 0.5 ml/kg/h for 5–6 and 8–9 year olds or < 500 mL/24-h for 13-17 year olds, and/or (4) If the timing of the collection was < 20 h or > 28 h	8	High
		167	56 (50%)	111	8–9	8.5 (0.5)				
		125	57 (55%)	103	13–17	14.6 (1.3)				
Martins [62]	2008	11	5 (45%)	11	2–4	3.7	Ibiá, Brazil [62]	Not described	2	Low
Maruhama [63]	Not described	11	6 (55%)	11	6–9.9	8 (1.3)	Not described	Not described	0	Low
		11	5 (45%)	11	10–18	14 (1.9)				
Matkovic [64]	Not described	381	0 (0%)	359	8–13	10.9 (7.7)	Ohio, United States	“Inappropriate urine collections identified by residual analysis after fitting a regression equation of 24-h creatinine excretion on LBM as obtained by dual-energy X-ray absorptiometry”	5	Medium
Melse-Boonstra [76]	1996	9	7 (100%)	7	6–9	8.1	Guatemala, Central America	Not described	2	Low
		13	13 (100%)	13	8–12	9.7	Benin, West Africa			
Mori [65]	2002	56	54 (46%)	54	6–11	8.1 (1.4)	Japan	Not described	3	Low
		364	0 (0%)	193	13–18	16.7 (1.3)				
Padrao [66]	2014	86	86 (100%)	86	7–11	8.7 (0.8)	Portugal	Creatinine excretion < 0.1 mmol/kg/day	6	Medium
		86	0 (0%)	86	7–11					
Rafie [67]	2015 –2016	128	42 (33%)	128	11–18	14.4 (2.0)	Isafahn, Iran	(1) Volume < 500 mL, and/or (2) Creatinine excretion < 0.1 mmol/kg/day	4	Medium
		129	57 (44%)	129	11–18	14.3 (2.2)				
		117	55 (47%)	117	11–18	14.6 (2.1)				
Rugg-Gunn [68]	Not described	27	27 (100%)	27	4–6	Not described	Dambulla, Sri Lanka	Not described	4	Medium
		26	0 (0%)	26	4–6					
		20	20 (100%)	20	4–6		Newcastle, England			
		24	0 (0%)	24	4–6					
Saied [37]	2015–2016	131	68 (52%)	131	6–18	9.8 (2.4)	Rabat, Morocco	Urine collections were considered incomplete and then excluded if (1) the volume is less than 300 ml; (2) more than a few drops of urine were reported lost during urine collection; (3) the collection time was outside of < 20 h or > 28 h; and (4) urinary creatinine was < 280 mg/l or > 2590 mg/l	9	High
Sinaiko [69]	Not described	40	20 (50%)	40	11–14	13.9 (1.3)	Minnesota, United States	Not described	1	Low
		243	122 (50%)	243	11–14	13.3 (1.6)				
Staessen [70]	1979–1981	82	82 (100%)	82	10–19	14.3 (3.0)	Belgium	“Urinary creatinine excretion was plotted against body weight after stratification for age and sex. Subjects who fell outside the two standard deviation range of the regression lines were excluded”	4	Medium
		78	0 (0%)	78	10–19	14.4 (2.6)				
Touitou [71]	Not described	9	9 (100%)	9	9–11	10.8 (0.11)	France	Not described	0	Low
Villa [72]	Not described	20	20 (100%)	20	3–6	4.4 (0.8)	Santiago, Chile	Not described	3	Low
Zhang [73]	2012	10	5 (50%)	16	2–11	10.0 (3.2)	China	Urinary creatinine used—cutoff not described	3	Low
Zohouri [74]	1995–1996	78	Not described	78	3–4.9	Not described	Fars Province, Iran	Creatinine excretion < 14 mg/kg/day or > 20 mg/kg/day	3	Low
Zohouri [75]	2002	7	5 (71%)	7	1–3	3 (0.6)	Newcastle, England	“Validated by measuring the urinary excretion of creatinine in each 24-h urine sample and comparing this with the standard reference value for creatinine excretion”	1	Low

*NOS* Newcastle–Ottawa Scale for cohort studies

*NOS rating based on score out of 10—low: 0–3; medium: 4–7; high 8–10

**Table 4:** Characteristics of studies considered for the primary analysis where 24-h urine volume was presented as median (IQR), *n* = 7 studies

Study	Study Year(s)	*n*	Male, *n* (%)	Valid urine samples	Age range (years)	Age, mean (SD) years	Country	Exclusion criteria for urine samples	NOS score	
									Score out of 10	Rating*
Campanozzi [81]	Not described	303	303 (100%)	303	6–9	10.1 (2.9)	Italy	Creatinine excretion < 0.1 mmol/kg/day [81]	6	Medium
		228	228 (100%)	228	9–11.3					
		235	235 (100%)	235	11.4–18					
		162	0 (0%)	162	6–9					
		180	0 (0%)	180	9–11.3					
		316	0 (0%)	316	11.4–18					
Degen [25]	1985–1989	69	35 (51%)	164	6–10	10.2 (*)	Dortmund, Germany	(1) Creatinine excretion < 0.1 mmol/kg/day (2) Samples reported or found to contain incomplete micturations	6	Medium
	1990–1994	70	35 (50%)	197	11–13	13.0 (*)				
Grases [82]	Not described	105	64 (61%)	105	5–17	12.0 (3.0)	Majorca, Spain	Not described	1	Low
Libuda [26]	2003–2009	15	15 (100%)	18	3–3.9	3.1 (*)	Dortmund, Germany	Creatinine excretion < 0.1 mmol/kg/day	5	Medium
		54	54 (100%)	83	13–14.9	14.0 (*)				
		66	66 (100%)	145	15–18	16.2 (*)				
		24	0 (0%)	25	3–3.9	3.2 (*)				
		56	0 (0%)	84	13–14.9	14.0 (*)				
		70	0 (0%)	144	15–18	16.2 (*)				
Montenegro-Bethancourt [28]	2000–2010	146	146 (100%)	269	4–6.9	5.0 (*)	Dortmund, Germany	(1) Creatinine excretion < 0.1 mmol/kg/day (2) Collection time < 20 h	5	Medium
		147	0 (0%)	264	4–6.9	5.0 (*)				
Montenegro-Bethancourt [29]	1996–2002	30	30 (100%)	30	13–18	15.0 (*)	Dortmund, Germany	(1) Creatinine excretion < 0.1 mmol/kg/day (2) No reported missed collections	6	Medium
		30	0 (0%)	30	13–18	14.0 (*)				
Montenegro-Bethancourt [30]	1993–2010	265	265(100%)	985	6–13	9.0 (*)	Dortmund, Germany	Creatinine excretion < 0.1 mmol/kg/day	6	Medium
		251	0 (0%)	974	6–13	9.0 (*)				

*NOS* Newcastle-Ottawa Scale for cohort studies

*NOS rating based on score out of 10—low: 0–3; medium: 4–7; high 8–10

Due to the wide age range of participants of the included studies, studies were grouped into three groups according to the age of the participants; 2–5 years (17 studies) [26, 28, 36, 43–45, 50, 51, 54–57, 60, 62, 68, 72, 74, 75], 6–12 years (20 studies) [25, 30, 35, 36, 45–48, 52, 54, 58, 59, 61, 63–66, 71, 76, 81] and 13 to < 19 years (12 studies) [25, 26, 29, 36, 49, 53, 54, 63, 65, 67, 69, 81]. These cut-points were chosen based on the WHO criteria for assessing population iodine deficiency, which defines school-aged children as between 6 and 12 years of age [6]. As some studies presented results broken down by age group, a single study may have been assigned to multiple age groups. Studies which crossed the age group cut-offs were assigned to the age group in which the majority of the participants would fall (e.g., a study with participants 9–13 years [59] would fall into the 6–12 year age group. Four studies [37, 70, 73, 82] which encompassed very large age ranges (i.e., > 8 years) were excluded from the sub group analyses using age cut offs.

#### Initial analysis

The overall mean [95% confidence interval (CI)] estimate of urine volume for all 44 studies was determined using a random effects model and presented for the group as a whole (i.e., 2–19 year olds), as well as broken down by age group.

#### Primary analysis: studies with ≥ 1 criterion for assessing the completeness of urine samples

As the inclusion of criteria for assessing the completeness of urine collections can result in the exclusion of over/under collectors, the primary analysis was limited to only those studies which reported at least one criterion for assessing the completeness of the included urine samples (*n* = 27, “Primary Analysis, Fig. 2). The overall mean (95% CI) estimate of daily urine volume was determined using a random effects model and displayed in forest plots, broken down by age group.

A one-way ANOVA was used to assess the differences in urine volume across the three age groups. In addition, a one-way ANOVA was used to assess differences in the urine volume between those studies which did not report any criteria for assessing the completeness of included urine samples and those which reported > 1 and > 2 criteria. Tukey’s post hoc tests were performed to determine significant differences between age subgroups. Heterogeneity was analysed using the *I*^2^ and *Q* statistics. The coefficient of variation (CV) of the mean volume for each age group was derived from the mean and SD random effects analysis and was calculated by dividing the SD for each age group’s urine volume by the mean and multiplying by 100. A two-sample *t* test used to assess the difference in volumes determined for the initial analysis compared to the primary analysis (i.e., limited to studies with > 1 criterion for assessing the completeness of the urine samples) across the three age groups. As 11 studies had presented the results broken down by gender [26, 28–30, 37, 53, 65, 66, 68, 70, 81], a two-sample *t* test was used to evaluate the difference in 24-h urine volume between genders.

To examine whether climate had an impact on overall urine volume, studies were classified according to climate based on their proximity to the equator. Studies which were conducted in countries which lie between the Tropic of Cancer and Tropic of Capricorn (23.5° north and south) (2 studies) [54, 55] were classified as having a “warm” climate, whereas studies which fell outside of this area were classified as having a “cold” climate (25 studies) [25, 26, 28–30, 35–37, 43, 46, 48, 52, 56, 57, 60, 61, 64, 66, 67, 70, 73–75, 81, 82]. A two sample *t* test was used to assess the difference in urine volume between climates.

## Results

### Summary of studies considered for primary analysis

The 44 studies considered for inclusion in the primary analysis were published from 1981 to 2018 (Tables 3 and 4). Of these, 14 reported findings from Europe [25, 26, 28–30, 46, 50, 56, 60, 66, 70, 71, 81, 82], seven from the United Kingdom [36, 48, 56, 57, 61, 68, 75], seven from Asia [49, 51, 52, 58, 65, 68, 73], four from North America [55, 64, 69, 76], three from South America [44, 62, 72], three from the Middle East [43, 67, 74], three from Africa [37, 54, 76], and one from Australia [35].

Twenty-five studies reported creatinine excretion [25, 29, 35–37, 43, 45, 46, 49, 52–54, 58, 61, 63, 64, 67, 69–71, 73, 75, 77, 79, 81], 17 as 24-h excretion (mmol/24 h, mg/24 h, g/24 h) [29, 35–37, 45, 46, 49, 52–54, 58, 63, 64, 69, 70, 73, 81], six as a ratio of creatinine to body weight (mmol/kg, mg/kg) [25, 61, 67, 71, 75, 77] and two as a ratio to urine volume [43, 79].

### Quality of studies considered for primary analysis

The criteria used to evaluate the completeness of the urine samples was inconsistent between studies, 18 studies did not provide any information on criteria used to assess the completeness of the urine samples [44, 45, 47, 49–51, 53, 58, 59, 62, 63, 65, 68, 69, 71, 72, 76, 82]. Of the 26 studies which reported their urine assessment criteria, 21 used creatinine excretion per kilogram of bodyweight [25, 26, 28–30, 35–37, 43, 46, 52, 54, 61, 64, 66, 67, 70, 73–75, 81], based on established age and gender-specific cutoffs [85]. The remaining five studies relied solely on urine volume [56, 57], the number of reported missed collections [48], the excretion of other nutrients (i.e., fluoride) [55], and paraminobenzoic acid (PABA) recovery as a measure of completeness [60]. Across all 26 studies, a total of ten studies utilised urinary volume as an indicator of completeness [35–37, 43, 48, 52, 56, 57, 61, 67], three [35, 37, 52] used the cut-off of 300 mL/24 h based on previously published criteria [86], two studies [56, 61] utilised the WHO criteria of < 5 mL/h and < 9 mL/h for < 6 and ≥ 6 year olds, respectively, and two studies [36, 67] in older children (13–19 years) used the cutoff of < 500 mL/24 h, based on previously published criteria [86]. The cut-offs used by the remaining three studies [43, 48, 55] were based on the distribution of volume in the sample, enabling the exclusion of extreme outliers (e.g., 4SDs below the geometric mean [48]). Of the 26 studies which reported the urine exclusion criteria, 6 did not report the number of urine samples excluded from the final analysis [25, 54, 73–75, 82].

As a result of the inconsistency in the criteria used to assess the completeness of the urine samples between studies, 23 studies (54%) scored low on the NOS quality scale [44–46, 49–51, 53–55, 57–59, 62, 63, 65, 69, 71–76, 82] (Tables 3 and 4). Eighteen studies (42%) were classified as “medium” [25, 26, 28–30, 43, 47, 48, 52, 56, 60, 61, 64, 66–68, 70, 81], and only three studies provided sufficient detail regarding the 24-h urine collection procedure to be classified as “high” quality [35–37] (Tables 3 and 4).

### Initial analysis

The overall mean urine volume estimate (95% CI) for all 44 studies (*n* = 7712, 9538 urine samples) was 722 (686, 758) mL/24-h. Eleven studies reported the results broken down by gender [26, 28–30, 37, 53, 65, 66, 68, 70, 81]. There was no difference in mean urine volume between genders (858.09 (286) mL/24-h (*n* = 2635, 2635 urine samples) and 818 (240) mL/24-h (*n* = 2504, 2504 urine samples), for males and females, respectively, *P* = 0.7). When broken down by age group, there were more than three times the number of urine collections for 6–12 year olds compared with 2–5 year olds and approximately half the number of samples for the 13–19 year olds. Sixteen studies reported results for 2–5 year olds (*n* = 1304, 1557 urine samples), 20 for 6–12 year olds (*n* = 3772, 5210 urine samples) and 12 for 13–19 year olds (*n* = 2230, 2359 urine samples). For each of the three age groups, the overall estimate (95% CI) was 461 (413, 509) mL/24-h among 2–5 year/olds (Supplemental Fig. 1), 758 (725, 791)mL/24-h for 6–12 year olds (Supplemental Fig. 2) and 1048 (973, 1123) mL/24-h for 13–19 year olds (Supplemental Fig. 3). There was a significant difference in the mean urine volume across the three age groups (*P* < 0.001).

### Primary analysis limited to studies with > 1 urine completeness assessment criterion

Twenty-six studies reported ≥ 1 criterion for assessing the completeness of the urine samples (*n* = 6322, 8331 urine samples) [25, 26, 28–30, 35–37, 43, 46, 48, 55–57, 60, 61, 64, 66, 67, 70, 73–75, 81]. The overall urine volume estimate (95% CI) for these studies was 773 (654, 893) mL/24-h [median (IQR) 737 (284) mL/24-h]. When studies were assessed by climate (“warm” climate (2 studies) [54, 55] versus “cold” climate (25 studies) [25, 26, 28–30, 35–37, 43, 46, 48, 52, 56, 57, 60, 61, 64, 66, 67, 70, 73–75, 81, 82]) there was no difference in mean (95% CI) 24-h urine volume: “warm” hot 788 (244, 1332) mL/24-h vs “cold” 779 (713, 845) mL/24-h, *P* = 0.96.

**Fig. 3:**
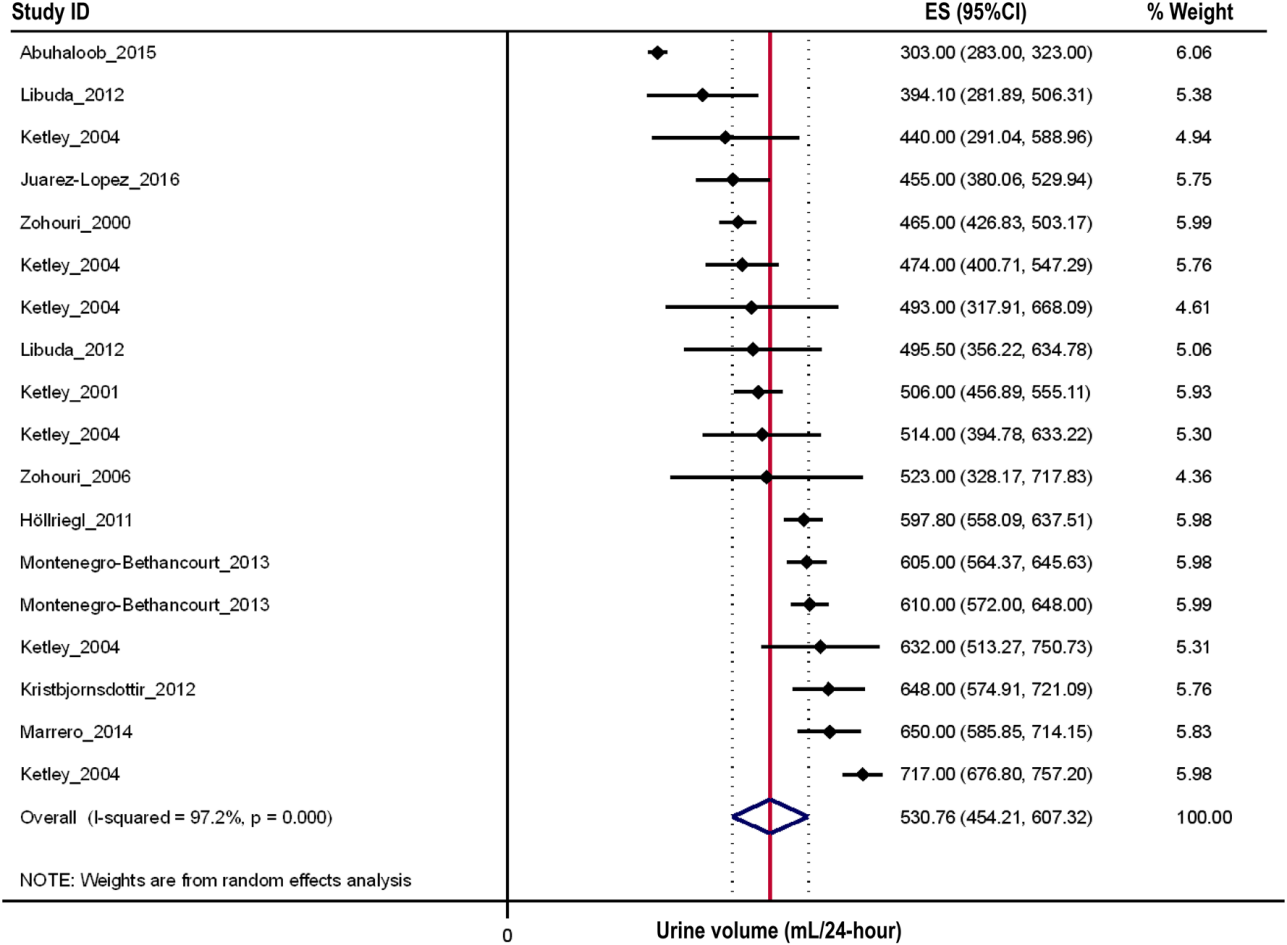
Forest plot of studies assessing 24-h urine volumes of 2–5 year olds with > 1 urine assessment criterion (*n* = 1084)

**Fig. 4:**
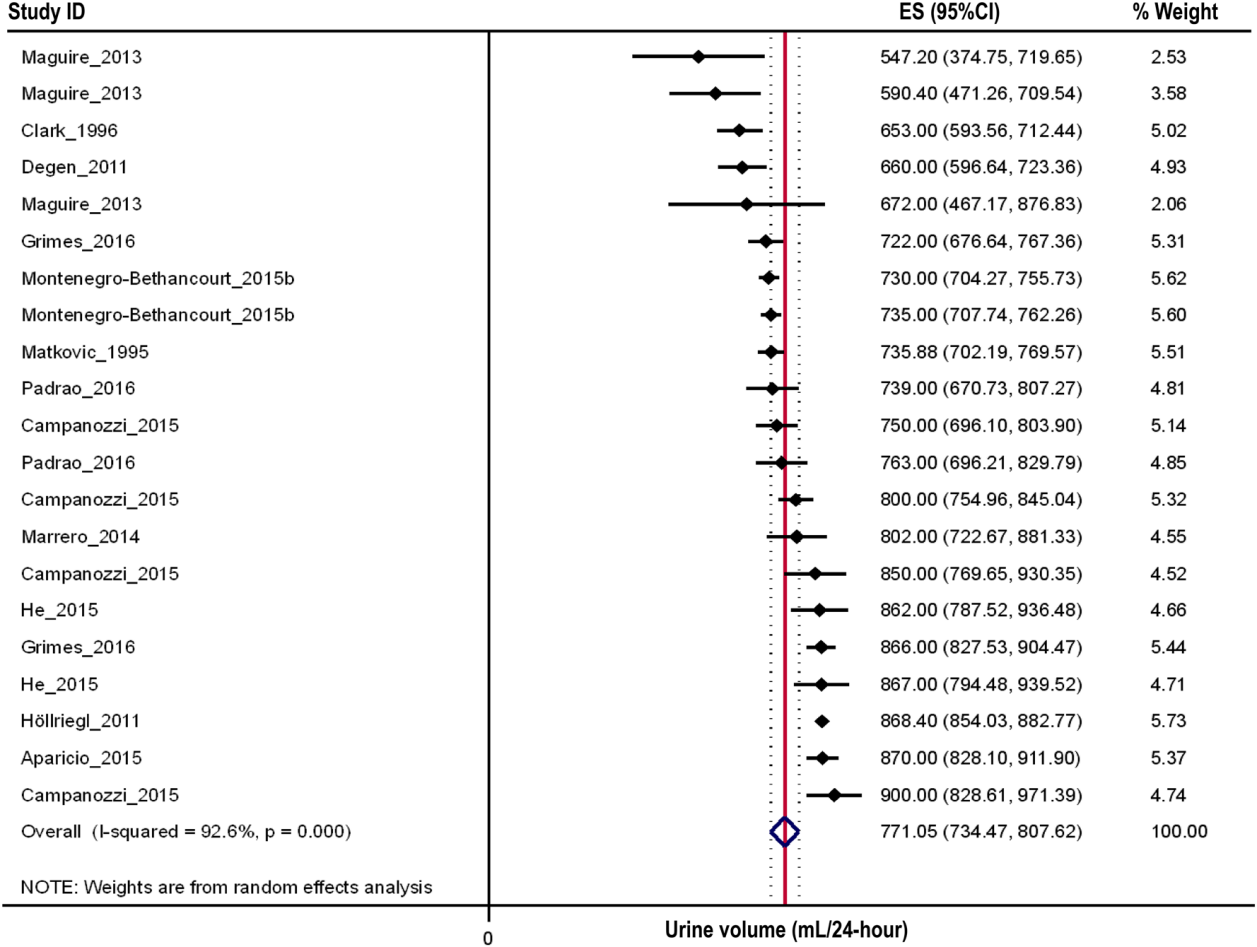
Forest plot of studies assessing 24-h urine volumes of 6–12 year olds with > 1 urine assessment criterion (*n* = 3628)

**Fig. 5:**
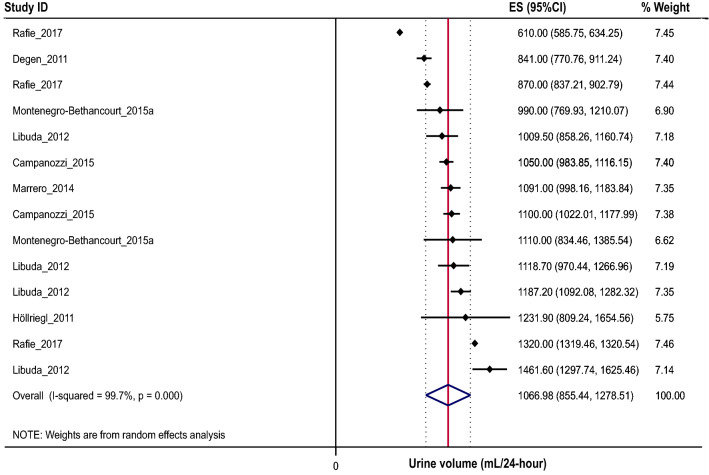
Forest plot of studies assessing 24-h urine volumes of 13–19 year olds with > 1 urine assessment criterion (*n* = 1438)

When broken down by age group, 11 studies reported results for 2–5 year olds (*n* = 987, 1240 urine samples) [26, 28, 36, 43, 54–57, 60, 74, 75], 12 for 6–12 year olds (*n* = 3596, 5038 urine samples) [25, 30, 35, 36, 46, 48, 52, 54, 61, 64, 66, 81] and seven for 13–19 year olds (*n* = 1438, 1746 urine samples) [25, 26, 29, 36, 54, 67, 81]. The overall estimate (95% CI) for each of the three age groups were 531 (454, 607) (Fig. 3), 771 (734, 808) (Fig. 4), and 1067 (855, 1279) (Fig. 5) mL/24-h, respectively. There was a significant difference in the mean urine volume across the three age groups (*P* < 0.001). Posthoc analyses revealed that children in the oldest age group had a 28% higher 24-h urine volume compared to those aged 6–12 years (1067 vs. 771 mL/24-h, *P* < 0.001) and approximately 50% higher urine volume compared to those aged 2–5 years (1067 vs. 531 mL/24-h, *P* < 0.001). Similarly, those aged 6–12 had a 31% higher volume compared to 2–5 year olds (771 vs. 531 mL/24 h, *P* < 0.001). There was significant between study heterogeneity across all three age groups (2–5 years/olds: *I*^2^ = 97.2%, *P* < 0.001, Fig. 3; 6–12 years/olds: *I*^2^ = 92.6%, *P* < 0.001, Fig. 4; 13–19 years/olds: *I*^2^ = 99.7%, *P* < 0.001, Fig. 5).

When comparing the mean urine volume between those studies which reported at least one criterion for assessing the completeness of urine samples and those which reported none, the only difference in mean urine volume was amongst the 2–5 year old age group. In this age group, the mean urine volume in the initial analysis (i.e., all studies were included) was 27% lower compared to the primary analysis (i.e., when the analysis was limited to only those studies with ≥ 1 criterion for assessing the completeness of urine samples (386 vs. 529 mL/24-h, *P* = 0.001).

Of the 26 studies with at least one criterion for assessing the completeness of the urine samples, only twelve [25, 28, 29, 35–37, 43, 48, 52, 55, 61, 64] had at least two urine criteria (*n* = 2867, 3191 urine samples). In these twelve studies, the mean (95% CI urine volume estimate was 742 (639, 844) mL/24-h. There was no difference in the mean (95% CI) urine volume estimate of these studies, compared to those with only one assessment criterion [*n* = 15, 798 (664, 932) mL/24-h, *P* = 0.33] or those with none [*n* = 18, 635 (577, 692) mL/24-hs *P* = 0.28].

For those studies with at least one criterion for assessing the completeness of urine collections there was less variation in daily urinary volume for those 6–12 years compared to 2–5 and 13–19 year olds. The co-efficient of variation (CV) for 6–12 year olds was 13% compared to 20% in 2–5 and 13–19 year olds. There was no difference in CVs between those studies with no reported criterion for completeness and those reporting at least one criterion (overall CV: 32% vs. 27%). In contrast, the CV for those aged between 13 and 19 years was reduced to 12% in those studies utilising least two criteria for assessing completeness of urine collection, compared with 20% for those studies utilising only one criterion for assessment for completeness.

## Discussion

This is the first study to systematically review the 24-h urine volume of children and adolescents. The overall 24-h overall urine volume estimate (95% CI) of 2–19 year olds was 778 (661, 895) mL/24-h urine. As expected, older children had higher urine volumes with children in the oldest age group (13–19 years) having a 28% higher 24-h urine volume compared to those aged 6–12 years (1067 vs. 771 mL/24-h, *P* < 0.001) and approximately 50% higher urine volume compared to those aged 2–5 years (1067 vs. 531 mL/24-h, *P* < 0.001).

As approximately 90% of ingested iodine is excreted in the urine within 24–48 h [87], current recommendations for assessing the severity of iodine deficiency within a population are based on the measurement of urinary iodine concentration (UIC), expressed as µg iodine per liter of urine, in random ‘spot’ urine samples collected from school-aged children (i.e., 6–12 years [6]). Although these recommendations were originally based on the observation that goiter prevalence was < 10% in populations of children and adults where the average daily iodine intakes were > 100 µg, later iterations extrapolated this value to represent a concentration, expressed as µg/L [4]. However, results from this analysis indicate that the average 24-h urine volume of school-aged children, the group commonly recommended for use in population iodine monitoring, is not 1 L and is closer to 0.8 L. Therefore, a median spot urine concentration of 100 µg/L (extrapolated to a median iodine intake of 100 µg/day) would overestimate iodine intake by approximately 30 µg/day.

Iodine excretion from spot urine samples is most often expressed as a concentration or as a ratio to creatinine excretion (I/Cr, µg iodine/g creatinine) [29, 88, 89]. The use of I/Cr to estimate daily iodine intake is believed to provide an accurate estimate of daily iodine excretion from spot urine samples as creatinine, an endogenous indicator of lean body mass is relatively constant from day to day in healthy adult populations [32, 90]. Whilst this is true in adults, estimating expected creatinine excretion values for children can be difficult as creatinine excretion can be affected by muscle mass, age, gender, ethnicity and onset of puberty [91]. Some equations for estimating daily creatinine excretion are able to account for these factors, whilst others provide a more crude estimate of daily creatinine excretion [92].

In addition, whilst the variation in individual iodine excretion between days is largely dependant on the iodine content of the diet, iodine excretion has also been found to vary over the course of the day in individuals [29, 88, 89, 92–97]. One study conducted in 42 adults and children (aged 4–60 years) found that urinary iodine excretion varied significantly by the timing of collection (*P* < 0.001), with lowest levels occurring in the morning and peaks observed following meals [98]. A recent systematic review of studies comparing spot and 24-h urine samples for estimating the iodine intakes of a population concluded that there is currently not enough evidence to determine whether iodine intake determined from spot urine samples provides an accurate reflection of daily iodine intake, as measured using 24-h urine samples [99].

Furthermore, whilst the WHO recommendation for assessing the iodine status of populations are primarily meant for use in school-aged children, and were derived primarily on data based on goiter prevalence estimates in school-aged children, they have also been used to define the iodine status of adult populations [100–103]. Issues concerning different urinary volume outputs among different subsets of the population and implications for iodine nutrition assessment have been previously identified by Zimmerman and Andersson [104]. They highlighted that as the urine volume of adults is closer to 1.5 L [27, 105], the use of the median UIC determined using spot urine samples could result in the underestimation of the iodine intakes of adult individuals within the population [104]. This was demonstrated in a recent study in 301 adults (18–64 years) from New Zealand, which compared median UIC from 24-h urine samples to the WHO criteria, both with and without adjustment for total urine volume [101]. This sample of adults was classified as iodine deficient using the WHO criteria, based on a median UIC of 73 µg/L. However, the measured 24-h UIE, which accounts for urine volume and averaged 2 L was closer to 127 µg/day [101]. This value is in excess of the 100 µg/day originally associated with reduced goiter prevalence [8] and would indicate that the iodine intakes of this group of adults may be sufficient [8]. The New Zealand study demonstrates the potential impact of not accounting for the daily urine volume may have on the assessment of iodine deficiency in populations when UIC determined from spot urine samples is used as a surrogate index of iodine intake.

### Strengths and limitations

We observed considerable between-study heterogeneity across all three age groups in the primary analysis limited to studies with > 1 indicator of urine completeness. For all three age groups the age range of participants included within each group varied considerably. For example, within the 2–5 year age group one study consisted of participants aged 1.5–3.5 years [56] whilst another consisted of participants aged 3–6 years [72]. Differences in both the age range of participants as well as the number of participants between studies, along with season of assessment and overall diet composition may have contributed to the observed heterogeneity.

In addition, there were considerable variations in the mean/median reported urine volumes, even within the three age groups, particularly for 2–5 year olds. This may represent the practical difficulties in obtaining accurate 24 h urine collections in young children, however, it is important to note that the included studies did not consistently report their 24-h urine collection protocol, nor the indicators used to assess the completeness of included urine samples. Of the 44 studies reviewed, 17 studies (40%) did not report at least one indicator for completeness of the 24-h urine samples. The only difference in the mean urine volume estimate between the total sample and those studies with ≥ 1 indicator for completeness of urine samples, was seen among the youngest age group (2–5 years) where average urine volume was 143 ml/24-h less in the studies which reported no criterion for assessing the completeness of included urine samples. A recent systematic review of methods for assessing the completeness of 24-h urine collections in adults and children (15–89 years) concluded that that the use of two or more indicators for assessing urine completeness increases the likelihood of detecting incomplete samples, thus increasing the validity of the results [33]. Our findings are contrary to this in that we found no difference in urine volume among those studies that had at least two urine assessment criteria, compared to those with only one. In the present review, only 12 studies utilised more than one criterion [25, 28, 29, 35–37, 43, 48, 52, 55, 61, 64], and there was no difference in mean 24-h urine volume estimated from these studies compared to the overall estimate from all 44 studies. However, there was less variation in daily urinary volume in the 6–12 year group (CV 13%). Overall there seemed to be little impact on urine volume variation of including a number of criteria for completeness of 24 h urine collection, except for the 13–19 year age group where studies that included at least two criteria for completeness appeared to have less variation (CV 16%) compared with studies with only one criterion (CV 20%).

In this analysis, only two studies collected 24-h urine samples from countries classified as having a “warm” climate”, compared to 25 studies from a “cold” climate. There is also considerable within and between person daily variability in iodine excretion [92, 95, 96, 98, 106, 107]. One study conducted in 42 adults and children (aged 4–60 years) observed that the lowest level of iodine excretion occurred in the morning with peaks observed following meals [98]. Furthermore, a study in adults noted that UIC determined from a fasting spot urine samples was 10% lower than that determined in a non-fasting spot urine sample [96]. Although this variation has yet to be assessed in children, this study indicates that the timing of a spot urine sample used to estimate the iodine intake of a population may also have a significant impact on the overall assessment of iodine nutrition. Therefore, it is clear that a number of factors need to be considered when making population estimates of iodine intake using spot urine collections across the age range from early childhood to adolescence.

## Conclusion

This is the first systematic review to report the average 24-h urine volume of children and adolescents from 44 studies representing 7712 individuals with 3772 individuals within the 6–12 year old age group, which included at least one criterion for completeness of urine collection. The average urine volume in this group was 771 mL, which is less than 1 L. This has implications when extrapolating median iodine values (µg/L) from spot urine samples to daily iodine intakes of 6–12 year old children as the average 24-h urine volume is less than 1 L, potentially resulting in an overestimate of dietary iodine intake in the order of 30%. Future studies employing spot urine samples to determine the iodine status of children and adolescents should consider undertaking 24-h urine collections in a subset of participants, to determine total urine volume and iodine excretion. This will allow the assessment of the accuracy of utilizing UIC as a proxy measure of daily iodine intake and potentially prevent the misclassification of iodine intakes in the population.

## Electronic supplementary material

Below is the link to the electronic supplementary material.

Supplementary material 1 (PDF 22 kb)

Supplementary material 2 (PPTX 68 kb)

Supplementary material 3 (PPTX 69 kb)

Supplementary material 4 (PPTX 62 kb)

## Abbreviations

DONALD: Dortmund Nutritional and Anthropometric Longitudinally Designed Study
NOS: Newcastle–Ottawa scale
PRISMA: Preferred Reporting Items for Systematic Review and Meta-Analysis
UIC: Urinary iodine concentration
UIE: Urinary iodine excretion
WHO: World Health Organization
