# Supplementary material for: A systematic review and meta-analysis of 24-h urinary output of children and adolescents: impact on the assessment of iodine status using urinary biomarkers
Source: Eur J Nutr. 2019 Nov 29;59(7):3113–31. doi: 10.1007/s00394-019-02151-w (PMC7501103; doi:10.1007/s00394-019-02151-w)
Supplement: Supplementary file 1 — Supplementary material 1 (PDF 22 kb) [file 394_2019_2151_MOESM1_ESM.pdf]

<u>NEWCASTLE-OTTAWA SCALE FOR ASSESSING THE QUALITY OF INCLUDED STUDIES</u>

A STUDY CAN SCORE A MAXIMUM OF 10 STARS

Selection (maximum 2 stars)

1) <u>Representativeness of the study sample (maximum 2 stars)</u>
    a. truly representative of the source population ☆☆
    b. somewhat representative of the source population ☆
    c. selected group of users eg. small age range, single gender, small sample size
    d. no description of the derivation of the cohort

Comparability (maximum 1 star)

1) <u>Normalisation of urine collection time</u>
    a. collection normalized to 24-hour period ☆
    b. some other normalization applied to results ☆
    c. results not normalized
    d. unclear as to whether results were normalized

Assessment of Outcome: 24-hour urine collection (maximum 7 stars)

1) <u>Instructions given to participants (maximum 2 stars)</u>
    a. clear instructions given to both the parents and the child participant ☆☆
    b. instructions only given to either the parent or the participant☆
    c. unclear as to the level of instruction given

2) <u>Start/stop time of urine collection (maximum 1 star)</u>
    a. parent or researcher recorded the start and stop time ☆
    b. child reported start and stop time
    c. no information provided

3) <u>Sample inclusion/exclusion criteria (maximum 4 stars)</u>
    a. time of collection ☆
    b. volume of collection ☆
    c. number of missed samples/spillages ☆
    d. creatinine cutoff ☆
    e. no description of urine exclusion/inclusion criteria
