# Supplementary material for: A systematic review and meta-analysis of 24-h urinary output of children and adolescents: impact on the assessment of iodine status using urinary biomarkers
Source: Eur J Nutr. 2019 Nov 29;59(7):3113–31. doi: 10.1007/s00394-019-02151-w (PMC7501103; doi:10.1007/s00394-019-02151-w)
Supplement: Supplementary file 4 — Supplementary material 4 (PPTX 62 kb) [file 394_2019_2151_MOESM4_ESM.pptx]

## Slide 1
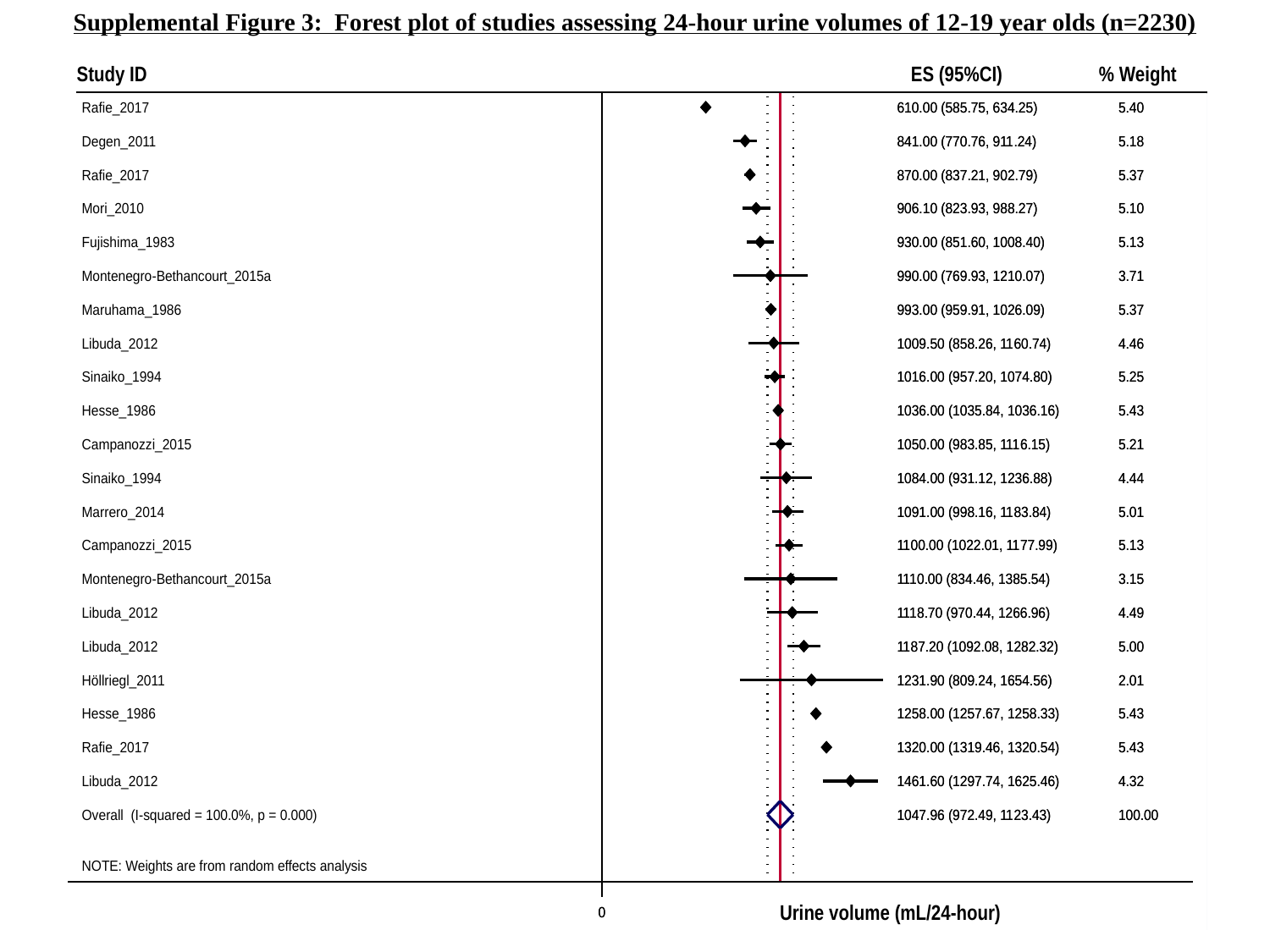

Supplemental Figure 3: Forest plot of studies assessing 24-hour urine volumes of 12-19 year olds (n=2230)
Study ID	ES (95%CI)	% Weight
	Urine volume (mL/24-hour)
